# ZooTraits: An R shiny app for exploring animal trait data for ecological and evolutionary research

**DOI:** 10.1002/ece3.11334

**Published:** 2024-04-29

**Authors:** Thiago Gonçalves‐Souza, Beatriz Milz, Nathan J. Sanders, Peter B. Reich, Brian Maitner, Leonardo S. Chaves, Gabriel X. Boldorini, Natália Ferreira, Reginaldo A. F. Gusmão, Phamela Bernardes Perônico, Fabrício B. Teresa, María Natalia Umaña

**Affiliations:** ^1^ Institute for Global Change Biology, School for Environment and Sustainability University of Michigan Ann Arbor Michigan USA; ^2^ Department of Ecology and Evolutionary Biology University of Michigan Ann Arbor Michigan USA; ^3^ Programa de Pós‐Graduação em Etnobiologia e Conservação da Natureza, Departmento de Biologia Universidade Federal Rural de Pernambuco Recife Brazil; ^4^ Pós‐graduação em Ciência Ambiental, Instituto de Energia e Ambiente Universidade de São Paulo São Paulo Brazil; ^5^ Department of Forest Resources University of Minnesota St Paul Minnesota USA; ^6^ Department of Geography University at Buffalo Buffalo New York USA; ^7^ Comparative BioCognition, Institute of Cognitive Science University of Osnabrück Osnabrück Germany; ^8^ Programa de Pós‐Graduação em Biodiversidade, Departmento de Biologia Universidade Federal Rural de Pernambuco Recife Brazil; ^9^ Programa de Pós‐Graduação em Recursos Naturais do Cerrado Universidade Estadual de Goiás Anápolis Brazil; ^10^ Laboratório de Biogeografia e Ecologia Aquática Universidade Estadual de Goiás Anápolis Brazil

**Keywords:** animal trait database, Open Science, Raunkiæran shortfall, trait‐based ecology

## Abstract

Animal trait data are scattered across several datasets, making it challenging to compile and compare trait information across different groups. For plants, the TRY database has been an unwavering success for those ecologists interested in addressing how plant traits influence a wide variety of processes and patterns, but the same is not true for most animal taxonomic groups. Here, we introduce ZooTraits, a Shiny app designed to help users explore and obtain animal trait data for research in ecology and evolution. ZooTraits was developed to tackle the challenge of finding in a single site information of multiple trait datasets and facilitating access to traits by providing an easy‐to‐use, open‐source platform. This app combines datasets centralized in the Open Trait Network, raw data from the AnimalTraits database, and trait information for animals compiled by Gonçalves‐Souza et al. (2023, *Ecology and Evolution* 13, e10016). Importantly, the ZooTraits app can be accessed freely and provides a user‐friendly interface through three functionalities that will allow users to easily visualize, compare, download, and upload trait data across the animal tree of life—*ExploreTrait*, *FeedTrait*, and *GetTrait*. By using *ExploreTrait* and *GetTrait*, users can explore, compare, and extract 3954 trait records from 23,394 species centralized in the Open Traits Network, and trait data for ~2000 species from the AnimalTraits database. The app summarizes trait information for numerous taxonomic groups within the Animal Kingdom, encompassing data from diverse aquatic and terrestrial ecosystems and various geographic regions worldwide. Moreover, ZooTraits enables researchers to upload trait information, serving as a hub for a continually expanding global trait database. By promoting the centralization of trait datasets and offering a platform for data sharing, ZooTraits is facilitating advancements in trait‐based ecological and evolutionary studies. We hope that other trait databases will evolve to mirror the approach we have outlined here.

## Graphical abstract

ZooTraits was developed to tackle the challenge of accessing trait datasets by providing an easy‐to‐use, open‐source platform. This app offers three functionalities that will allow users to easily visualize, compare, download, and upload trait data—ExploreTrait, FeedTrait, and GetTrait

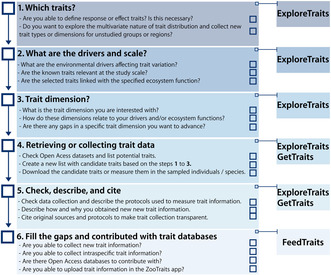

## 1 INTRODUCTION

The rapid growth of data availability in ecology and evolution has opened up new opportunities for researchers to advance and improve critical theoretical, conceptual, and applied aspects of both disciplines. In recent decades, functional trait approaches have become increasingly popular in various scientific disciplines due to their ability to provide a more mechanistic understanding of communities and their potential for generalization across different scales and systems (de Bello et al., 2021; Enquist et al., 2015; McGill et al., 2006; Reich et al., 1997; Wright et al., 2004). These approaches involve measuring functional traits, defined as characteristics of organisms that influence their fitness (Arnold, 1983; Violle et al., 2007). While most studies have primarily focused on plants (Green et al., 2022), there has been a growing interest in expanding the use of functional traits to animals and other organisms, incorporating new traits that capture movement and behavior and their potential response to environment or impacts on ecosystem functioning (e.g., Raffard et al., 2017).

Over the years, researchers have accumulated vast amounts of trait measurements, resulting in hundreds or even thousands of records for multiple species across different systems. Scientists have started compiling large databases to capitalize on this wealth of information, such as the BIEN (Enquist et al., 2016; Maitner et al., 2018) and TRY (Kattge et al., 2011) databases that are focused on plant traits. More recently, other initiatives such as OpenTraits have compiled trait information on different organisms like plants, animals, and fungi (https://opentraits.org, see also Oliveira et al., 2017; Parr et al., 2017; Tobias et al., 2022; Wilman et al., 2014). These databases have proven invaluable in addressing questions at broad spatial scales, including trait–trait relationships (Díaz et al., 2016), trait–environmental relationships (Bruelheide et al., 2018), and the role of traits mediating competition (Kunstler et al., 2016). For example, since its origin in 2007, the TRY database has gained a community of approximately 5000 users submitting over 1000 requests annually. Plant‐related studies have benefited from these centralized trait hubs, which offer advantages in trait selection (Kattge et al., 2020).

Despite the growing ubiquity of these databases in macroecological research—including studies that examine trait–environment, trait–ecosystem, or trait–trait relationships—collecting and assessing trait information has some acknowledged limitations (Herberstein et al., 2022; Keller et al., 2023). For example, many datasets have a limited taxonomic scope or focus solely on species from specific geographical regions. In some instances, the raw data are accessible only through meticulous searches into the supporting materials from published papers (Keller et al., 2023). This task becomes even more laborious for studies involving multiple taxonomic groups, as there is often a wide array of traits used among these groups (Gonçalves‐Souza et al., 2023). Consequently, researchers face difficulties in navigating and using these datasets effectively, highlighting the need for a solution. Additionally, finding suitable datasets is challenging when they are not centralized for a certain taxonomic group (e.g., Tobias et al., 2022 for birds) or in a central trait hub (Herberstein et al., 2022; Keller et al., 2023).

To overcome the above limitations, we have developed a user‐friendly shiny app called *ZooTraits*. This app serves as a tool to summarize information on traits across several animal taxonomic groups, ecosystems, and geographic regions to integrate multiple datasets centralized in the Open Trait Network to facilitate both the use of trait and contribution of trait data. The ZooTraits app provides: (i) access to species‐level trait data accessible for over 23,000 species from 20 datasets included in the Open Trait Network, and raw data for ~2000 species from the AnimalTraits (Herberstein et al., 2022), (ii) a possibility to continuously update the dataset with newly published research that will stimulate the development of a global list of traits used in several taxonomic groups, (iii) a global overview of traits used in animal ecology based on a dataset compiled by Gonçalves‐Souza et al. (2023). This tool can help researchers explore a wide range of traits and identify traits with a wide coverage in terms of number of groups and distribution of this information across ecosystems and geographical areas. Importantly, ZooTraits is not a *data* aggregator (e.g., unlike TRY, BIEN, or GBIF) but a *metadata* aggregator: it provides information about datasets and a pathway to accessing data from some, but does not necessarily provide access to all datasets, as many are under embargo or are not open access. Thus, by focusing on metadata, we require less storage space, allow data providers greater control over their datasets, and can include datasets which cannot be accessed due to licensing restrictions. We hope this tool not only fosters comparisons within and across taxonomic groups that help to identify general patterns of trait variation but also contributes to increased transparency and widening the accessibility to trait information. Importantly, as this app only streamlines the process of acquiring trait information, trait selection must be linked to best practices recommended by previous studies (e.g., Gonçalves‐Souza et al., 2023; Keller et al., 2023; Palacio et al., 2022), which suggest that trait selection should be mainly driven by biological hypotheses rather than solely relying on the data that are available.

## 2 DATA SOURCES AND COLLECTION METHODS

The ZooTraits app integrates different functionalities to explore, visualize, and download animal trait information. The data sources used to support the app were obtained from the Open Trait Network, the AnimalTraits (Herberstein et al., 2022), and a systematic review performed by Gonçalves‐Souza et al. (2023). These data sources used different collection methods and provided distinct trait information that were used to support the functionalities presented below.

The Open Trait Network (https://opentraits.org) is a global community of volunteer researchers that has centralized a trait hub with 20 datasets as of March 2024. Currently, it provides species‐level information on 3954 trait records from 23,394 species of invertebrates and vertebrates (Table 1). This information is not the actual trait data for a given species. Instead, the Open Trait Network offers trait names associated with these species (when available in the original dataset) and a URL containing all necessary information, which allows users to download the raw data for the selected species.

**TABLE 1:** Summary of the number of traits and species per phylum available from the Open Traits Network.

Phylum	Number of traits	Number of species
Chordata	1484	19,707
Arthropoda	745	1907
Cnidaria	229	1203
Mollusca	701	437
Echinodermata	125	42
Annelida	217	36
Platyhelminthes	90	14
Bryozoa	80	13
Ctenophora	45	10
Nematoda	37	5
Porifera	6	5
Brachiopoda	20	3
Tardigrada	21	3
Nemertea	40	2
Phoronida	22	2
Rotifera	27	2
Chaetognatha	25	1
Gastrotricha	19	1
Xenacoelomorpha	21	1

*Note*: By using the GetTrait capability, ZooTraits users may get the links to access the raw data for all taxonomic groups or a specific subset.

The AnimalTraits is a curated trait database that includes 2032 species from different taxonomic groups, including arthropods, mollusks, and tetrapods (Herberstein et al., 2022). Unlike the Open Trait Network, the raw data are directly available and, thus, can be directly downloaded from the ZooTraits app.

Lastly, we used the results from Gonçalves‐Souza et al. (2023), who compiled trait information from 1655 manuscripts that used functional traits in animal ecology. Different from the first two databases (i.e., Open Trait Network and AnimalTraits), that review paper provided a general overview of traits used in ecological studies across the Animal Kingdom including Vertebrata, Ecdysozoa, Protostomia, Spiralia, and Echinodermata from all continents and most marine biogeographical realms. Specifically, it summarized the following aspects: (i) the taxonomic level (e.g., species or genus level) used to collect trait information, which can be retrieved by downloading the raw data from the required taxonomic group; (ii) trait type (response or effect trait) and existence of intraspecific trait variation (yes or no); (iii) trait (or niche) dimension (defined by Winemiller et al., 2015): trophic, life history, habitat, defense, metabolic, other (undefined traits not included in the previous dimensions) or undetermined morphological traits (i.e., those morphological traits obtained by the authors of the primary papers but not included as relevant for any niche dimension); (iv) ecosystem type: terrestrial, freshwater or marine; and (v) geographical extent (local, regional, or global) and region (zoogeographical or marine biogeographical realm).

Taken together, these three data sources (i.e., Open Trait Network, AnimalTraits, and Gonçalves‐Souza et al., 2023) provide complementary information about trait use in animals and they will be used to support different functionalities of the ZooTraits app, as described below.

## 3 OVERVIEW OF THE SHINY APP FOR EXPLORING ANIMAL TRAIT DATA

Shiny is a framework that creates interactive web tools using R (Wickham, 2021). ZooTraits is an open‐source project (available on a GitHub repository at: https://github.com/thiago‐goncalves‐souza/zootraits), and it was organized based on three different functionalities that will allow users to display, visualize, access, and contribute with new trait information for animals:

1. *ExploreTrait*: Through this functionality, users can explore distribution and abundance/coverage of trait data information from almost ~1700 journal articles (at present, more later). This allows for investigating trends in trait variation and distribution across regions/taxonomic /habitats, etc. groups and identifying gaps in traits used across several animal taxonomic groups (reviewed in Gonçalves‐Souza et al., 2023). The app facilitates users to filter, sort, and analyze data by taxonomic groups, traits, habitats, ecosystems, scale, and geographical regions.
2. *FeedTrait*: through this functionality, users can upload/enter information associated with trait data, such as taxonomic level, trait type, etc. (see section 2), while supplying data to the *ExploreTrait* function. The upload of information is a continuous and necessary process that will keep the database updated. Data quality will be revised following a workflow (Figure S1) that will ensure data quality. The different versions (when new datasets are uploaded) will be preserved on GitHub and users will have the ability to obtain data from specific versions if needed.
3. *GetTrait*: through this functionality, users can access two openly available trait datasets as of March 2024, the Open Trait Network and the AnimalTraits (Herberstein et al., 2022).

The ZooTraits app will give users the option to download and/or upload raw data as *.csv file, as well as the ability to combine information from other publicly available trait datasets that let users download trait information for numerous animal taxonomic groups.

### 3.1 Installation

The ZooTraits shiny app does not require installation. The user can connect to the address https://ecofun.shinyapps.io/zootraits/ to use all functionalities. However, it is necessary to have an internet connection to use the app.

### 3.2 *ExploreTrait:* Exploring animal functional trait data

The default display of the *ExploreTrait* app enables users to visualize and contrast the key findings from every taxonomic group provided in Gonçalves‐Souza et al. (2023). The app will regularly update trait‐associated information using data contributed by users from newly published articles (details in section 3.3). Basically, its primary function is to filter, sort, and analyze data by taxonomic groups, traits, habitats, ecosystems, scale, and geographical regions.

**FIGURE 1:**
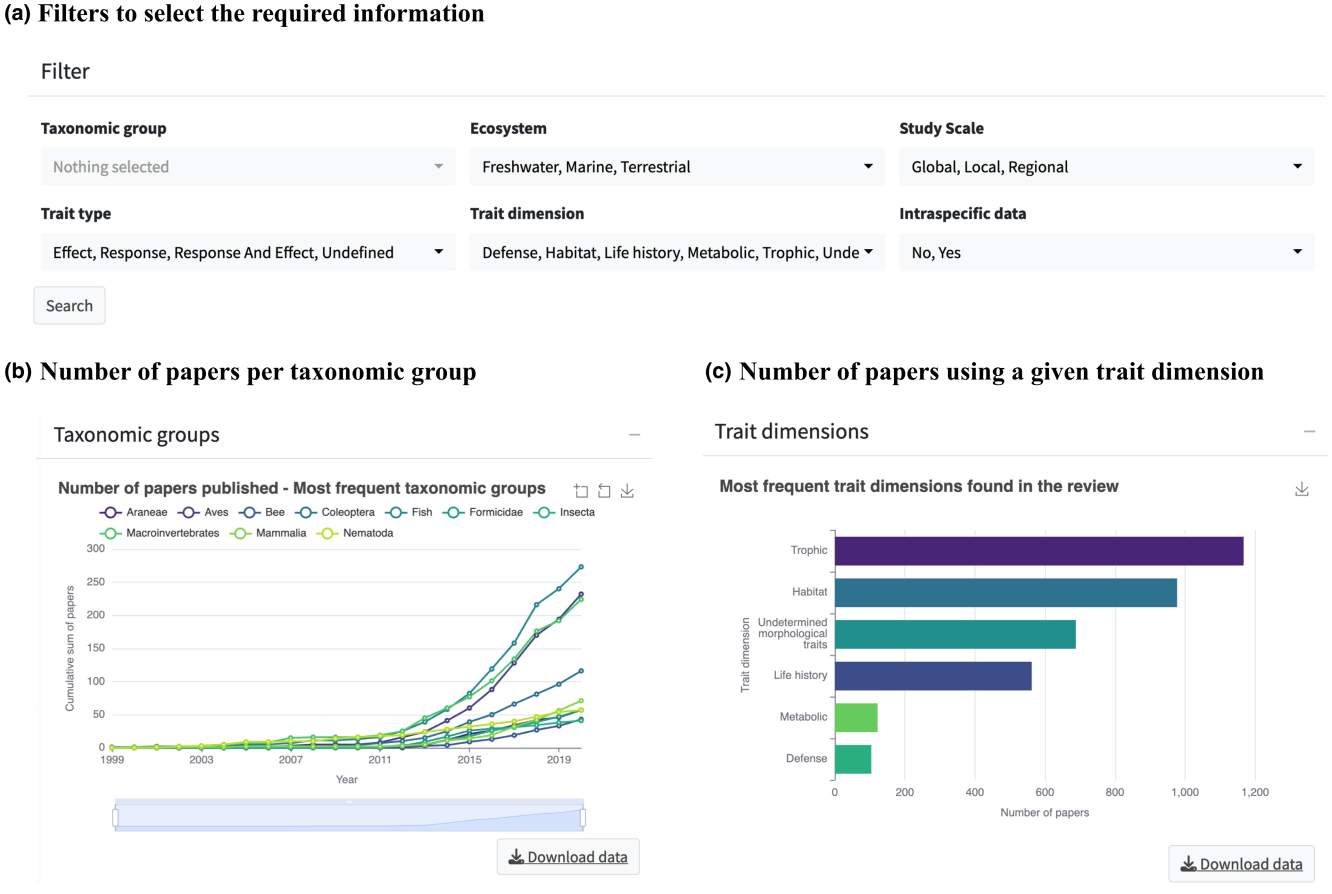
Screenshots of the Zoo App website, ExploreTrait functionality. (a) Filters that facilitate the selection of desired subset datasets based on taxonomic group, ecosystem, study scale, trait type and dimension, and/or intraspecific trait variation studies. Upon applying these filters, bar plots will be generated to summarize the number of papers (b) per taxonomic group and year, and (c) the most frequent trait dimensions.

Upon opening the app, the default display allows users to access basic descriptive trait information that can be filtered using different criteria in the ‘Data Exploration’ tab (Figure 1). These filters include (1) taxonomic group, (2) ecosystem (freshwater, marine, and terrestrial), (3) study scale (local, regional, and global), trait type (studies that used response and/or effect traits, or studies that did not define which trait type was used), (4) trait dimension (trophic, life history, habitat, defense, metabolic, other undefined traits not included in the previous dimensions or undetermined morphological traits), (5) and whether the study included or not intraspecific variability when measuring species traits.

**FIGURE 2:**
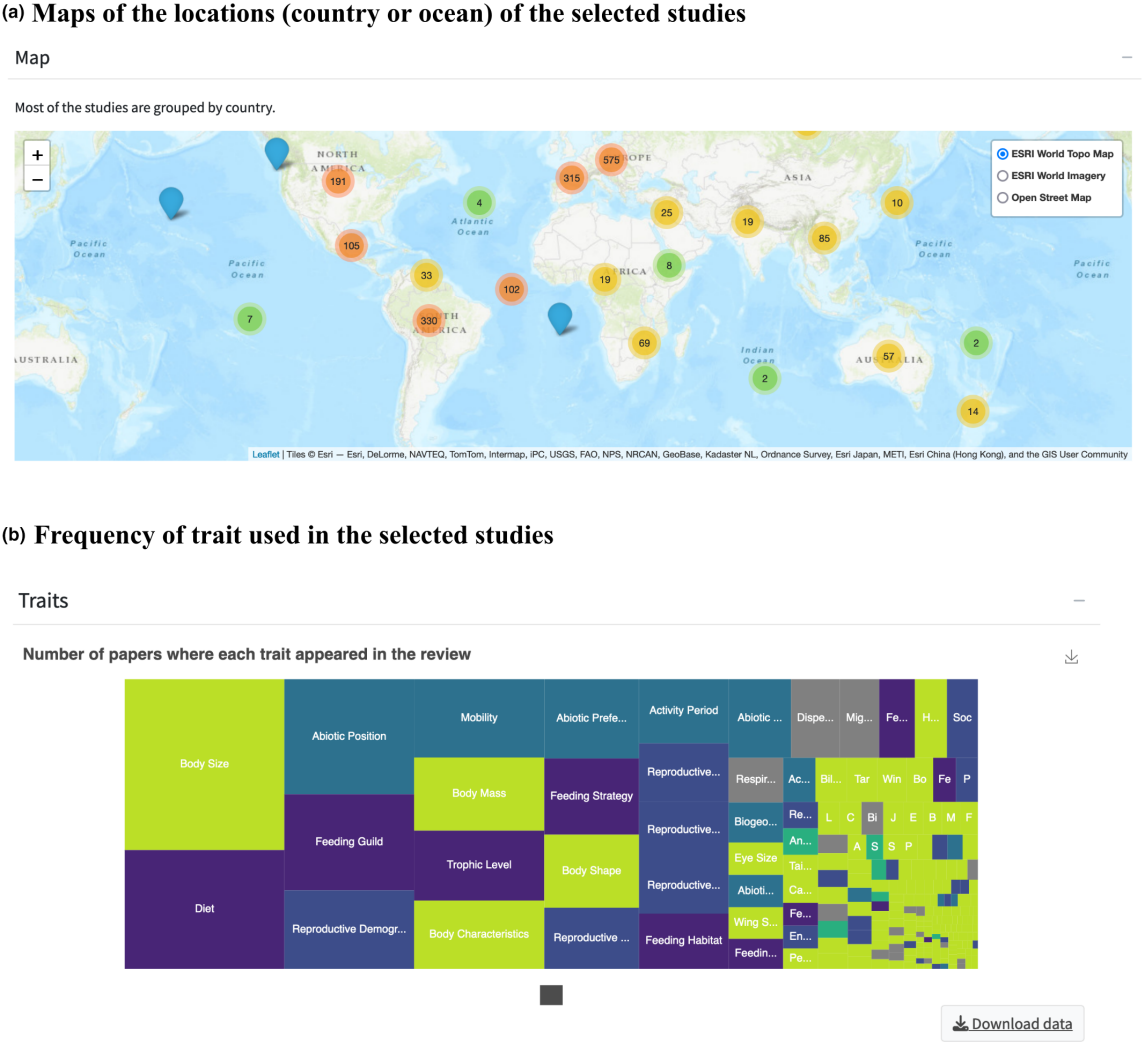
Screenshots of the ExploreTrait functionality after applying the filters (Figure 1a) showing (a) a map displaying the number of studies per nation or ocean and (b) a treemap displaying the frequency of traits in the chosen studies.

The *ExploreTrait* functionality accommodates various research needs. For example, users can retrieve information about the variety and frequency of traits used for a particular taxonomic group in the tab “Taxonomic group” and then select one or multiple groups listed in the options (Figure 1a). As a result, the app will display the number of papers per taxonomic group by year (Figure 1b), the most used trait dimensions (Figure 1c), a map with studies aggregated by continent (Figure 2a), and a treemap listing trait names and their frequencies (Figure 2b). Additionally, users have the option to download a list of the references and trait information for all studies within the selected filter by going to the “Dataset download” at the bottom of the Data Exploration window. To select a subset of the dataset, the user should click on the first floating panel (Figure 1a) and select one or several taxonomic groups. The user can also select a specific ecosystem, trait dimension, or study scale. After filtering the required subset, the user will be able to visualize four figures (insets in Figures 1 and 2) and download raw data in the “Dataset download”. The functionality also allows easy navigation on the map and access to linked DOI for each published study. Furthermore, we provided a “Metadata” tab to describe all variables in the dataset.

A relevant aspect of this app is the potential to extract data for different taxonomic groups. This capability can prove invaluable for future studies involving multiple organisms, helping researchers to select potential traits based on their (dis)similarities, frequency, distribution, and availability.

### 3.3 *FeedTrait:* Upload animal functional trait to the *ExploreTrait* functionality

As described above, at present, the *ExploreTrait* functionality directly relies on data obtained from Gonçalves‐Souza et al. (2023) to display and allow downloads of trait information. To complement this function, the *FeedTrait* functionality facilitates regular updates to the data displayed in the *ExploreTrait* function/tool.

**FIGURE 3:**
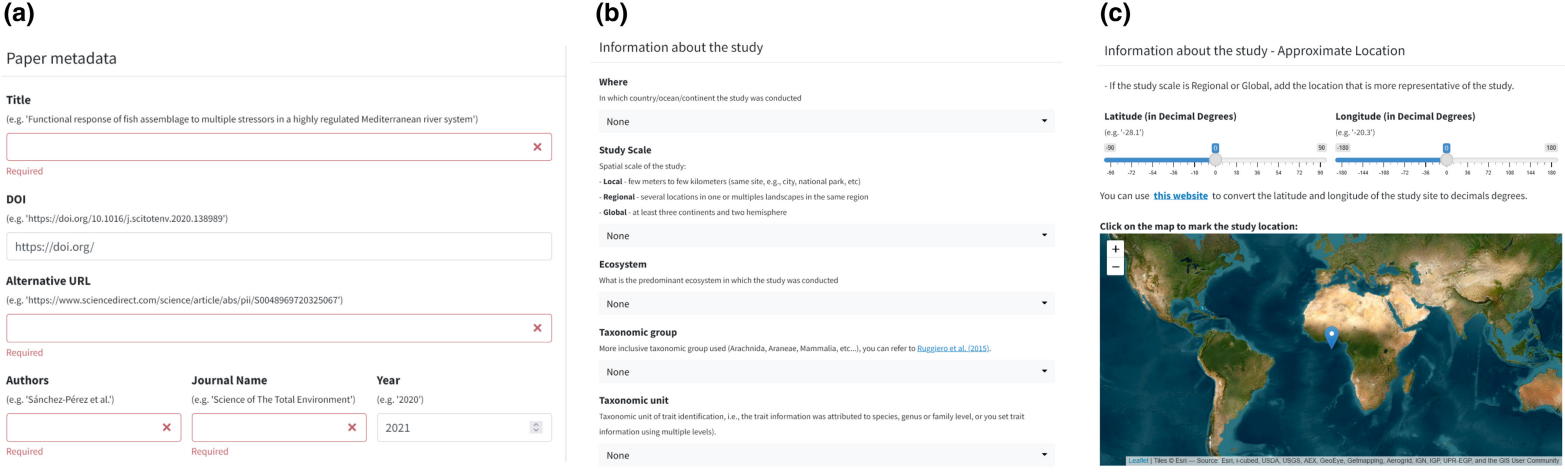
The FeedTrait functionality allows the user to upload trait information in two distinct forms. This screenshot depicts a user‐friendly option in which users can manually add information for a specific taxonomic group using the same metadata (manuscript title, DOI, first author, journal name, year, location, study scale, ecosystem, taxonomic group, trait type, trait dimension, and others) as the ExploreTrait. For studies performed in several locations, the user can upload a *.csv file with the same dataset as the manual method and the ExploreTrait. Required information is represented by fields in red.

To contribute data to *FeedTrait*, users can easily upload data using online form or a spreadsheet template (in a *.csv format) containing the following information: (1) DOI of the published study, (2) taxonomic group, (3) geographical region(s) and ecosystem(s), (4) trait type and dimension, and whether intraspecific trait data were considered or not (Figure 3). Each new row in the dataset should represent a newly published article, and the columns will provide a range of selectable options. This design ensures that data entry is efficient and minimizes the potential for input errors. For studies with multiple taxonomic groups, each row should represent a taxonomic group from that study.

The *FeedTrait* functionality is intended to foster a global network of open data to maintain a comprehensive registry of animal traits used in ecological and evolutionary studies across different regions and ecosystems. Therefore, *FeedTrait* is a repository for collaboration that welcomes participation of scientists around the world. Further, ZooTraits improves data discoverability, potentially making registered datasets more likely to be cited and thereby benefiting researchers who contribute.

We will ensure data quality by using a workflow connecting users and the ZooTraits team (Figure S1). This quality assurance/quality control (QA/QC) will follow user data enter → ZooTraits team quality control → ZooTraits app updates and version control on GitHub. Because of these data quality curation steps, we ensure data quality by asking (if necessary) for corrections in the databases before feeding the app. This is a particularly relevant process for those users that upload data by submitting a *.csv file.

### 3.4 *GetTrait* : Centralized tool to retrieve trait information from open‐access databases

The *GetTrait* functionality directly accesses freely available trait information in several open databases centralized in the Open Trait Network (OTN, https://opentraits.org/). The OTN currently includes 20 open datasets from several taxonomic groups within the Animalia Kingdom, such as Chordata, Arthropoda, Cnidaria, Porifera, and Rotifera (Table 1). As described earlier, AnimalTraits is a trait database including arthropods, mollusks, and tetrapods (Herberstein et al., 2022). While users can directly access the raw data from AnimalTraits, they will only be able to access links to datasets centralized in the OTN. Due to this distinction, the initial choice a user will make is the selection of the dataset.

After selecting the dataset, the user can search for data using Phylum, Class, and Order to select all available trait datasets for a specific taxonomic group. For example, if you are interested in studying spiders, you need to select the Phylum Arthropoda, Class Arachnida, and the Order Araneae in the “Filter” tab and then click on the “Search” tab. This command will retrieve a table with all spider species and traits available in the app. The user can download a *.csv file containing information on the family, genus, species, trait, and two links, one to taxonomic information from the catalog of life and the other to the original raw data (column ‘Dataset’). This column will redirect the user to the metadata of the specific dataset where that trait information can be retrieved, which includes links to the original paper and raw dataset. Moreover, the AnimalTraits database provides the actual trait value for different traits (e.g., body mass, brain size) and species.

### 3.5 Future capabilities

There are numerous capabilities that can be easily implemented in future versions of this app: (1) Direct access to raw trait information from several sources. Instead of redirecting users to the source datasets (e.g., AVONET, Tobias et al., 2022) by using the *GetTrait*, the user will be able to type the taxonomic group of interest and download a *.csv file containing all known species and their traits from the databases associated with that taxonomic group. (2) A potential *FeedTrait* 2.0 will allow users to add traits from individual species, which can feed not only the *ExploreTrait* (current version) but also the *GetTrait* functionality. (3) We plan to translate versions of the ZooTraits into other languages in order to decrease the barrier to submission for users who cannot read or write English. (4) AI tools will be used in QA/QC to improve the interaction between user‐supplied data, quality control, and app updates.

## 4 DISCUSSION AND CONCLUSION

The ZooTraits app is an open‐source ShinyApp with three functionalities that will let users visualize, compare, contribute, and download trait data for various taxonomic groups in the Animal Kingdom. This app is a user‐friendly web interface that integrates author‐provided datasets (*FeedTrait*) and global trait information (through the *ExploreTrait* and *GetTrait*). As of March, 2024, it extracts trait information from 1790 datasets (Gonçalves‐Souza et al., 2023), and 3954 trait records from 23,394 species (centralized in the Open Traits Network) of Chordata, Ecdysozoa, Protostomia, Spiralia, Cnidaria, Porifera, Echinodermata, Ctenophora, and other Phylum, and ~2000 species from the AnimalTraits database. The ZooTraits app allows comparisons of traits (e.g., trait type, trait niche dimension) used within and across taxonomic groups, geographical regions, and ecosystems. We posit that there are some benefits for ecological and evolutionary studies using traits accessed in this fashion. We also provided guidance on trait selection and the future directions we envision for trait‐based approaches.

First, the ZooTraits app serves as a tool for detecting trends, gaps, and biases in the selection of traits within and across various taxonomic groups, geographical regions, ecosystems, and trait dimensions. For example, if you are interested in understanding the global drivers of functional diversity in birds, you can use two different functionalities of the ZooTraits. The ExploreTrait functionality allows you to get trait information performed on all continents. By combining the visual exploration from the *Data Exploration* tab and the detailed information from the *Table* tab, you could detect that there are: geographical biases in the published literature on functional traits in birds, mostly focused in Europe and Americas; most studies focused on habitat and trophic dimensions, as well as morphological characteristics, but other dimensions, such as metabolic and defense traits, were overlooked. Furthermore, one can see the most used trait names in these studies, which can help identify the traits that could be easily compared across geographic regions, or taxonomic groups.

Additional benefits of this app included: it can identify the traits used at different scales, geographical regions, and ecosystems, and determine if certain traits are consistently employed across studies (Gonçalves‐Souza et al., 2023). This can be beneficial for studies of specific taxonomic groups seeking to standardize trait use and selection. Furthermore, it may act as a catalyst for stimulating future studies by identifying gaps in where studies of animal traits have been conducted for a given taxonomic group or trait dimension in a specific region (see, e.g., Maitner et al., 2023). Users can then combine this app‐extracted information with existing protocols (e.g., Moretti et al., 2017; Palacio et al., 2022; Tobias et al., 2022) to develop a reproducible methodology for trait selection. This app promotes data sharing, as users can access information of the compiled datasets for numerous species and traits from all world regions, and share their own data to facilitate the growth of this app. ZooTraits app will be an open‐source tool, simplifying the integration of existing databases (e.g., Open Trait Network, AnimalTraits), a task that is currently challenging (Keller et al., 2023). Lastly, the app can be utilized in field courses as a tool to expedite trait collection to support student in‐field projects that test ecological hypotheses. Furthermore, it can serve as a helpful guide for in‐class activities in thematic areas such as functional ecology, biodiversity and ecosystem functioning, and global change biology.

**FIGURE 4:**
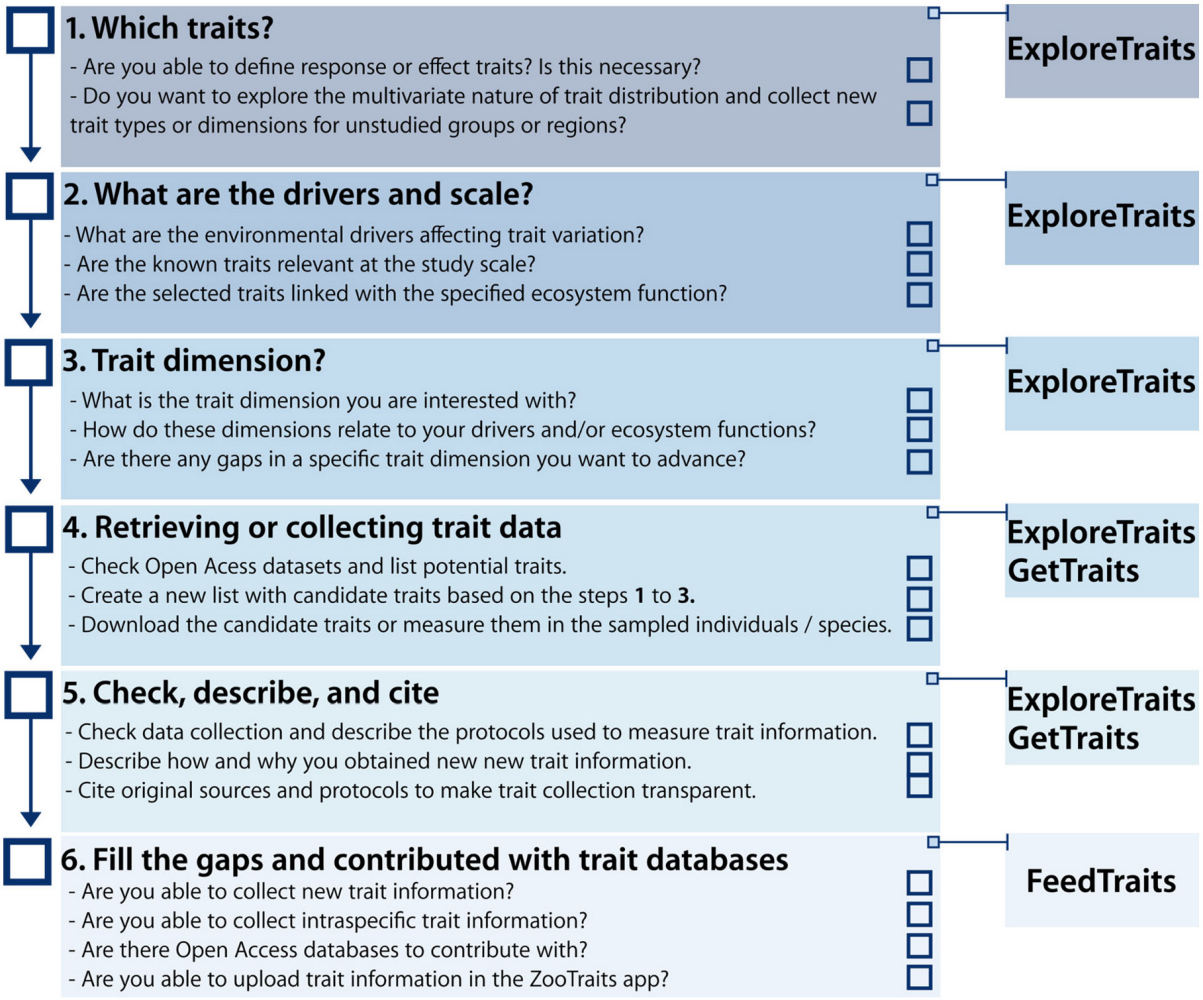
Framework to connect best practices in trait collection and the ZooTraits app. Each square can be checked by users to help explore all alternatives to a best trait sampling strategy.

Importantly, any reuse of trait data obtained from the app should be driven by the ecological/evolutionary question at hand (Gonçalves‐Souza et al., 2023; Keller et al., 2023; Palacio et al., 2022; see Figure 4). That is, ZooTraits users will be able to integrate the information retrieved from the app with their research questions/objectives to decide the best traits to use. For example, the user may ask the following questions to guide trait selection (Figure 4): which traits and trait dimensions are relevant and broadly used across taxonomic groups, ecosystems, and geographical regions? What are the drivers of trait variation and the scale in which the selected traits are relevant? We recommend that users of the ZooTraits app integrate trait selection with guidance from either taxon‐specific (Moretti et al., 2017; Tobias et al., 2022) or general frameworks (Damour et al., 2018; Gallagher et al., 2021; Kattge et al., 2020; Keller et al., 2023; Rosado et al., 2013, 2016; Weiss & Ray, 2019). This integration is needed to advance trait‐based ecology, especially for understudied taxonomic groups, since the selection of traits can significantly influence study conclusions (Lefcheck et al., 2015; Zhu et al., 2017). We provide guidance (Figure 4) for integrating prior recommendations (Gonçalves‐Souza et al., 2023; Keller et al., 2023) with the ZooTraits app to improve trait selection and data sharing for the future of global trait databases. One important clarification about the app is that it does not track changes in nomenclature, so we suggest users use tools and websites available to verify taxonomic names before using our app. We offered a non‐exhaustive list in Table S1 (see also Grenié et al., 2023).

The use of open‐source ShinyApps has seen significant growth in stimulating teaching and research activities in the fields of ecology and evolution in the past 5 years (Kass et al., 2023; McGuire et al., 2022; Segrestin et al., 2021; Weigelt et al., 2022). However, when it comes to trait‐based ecology, a key challenge lies in the knowledge shortfalls that hinder our ability to select appropriate traits or access information for certain taxonomic groups or geographical regions (Gonçalves‐Souza et al., 2023; Maitner et al., 2023). Furthermore, previous authors argued that it is critical to investigate multiple trait dimensions since studies on animals and plants have mostly concentrated on a restricted number of dimensions (Gonçalves‐Souza et al., 2023; Maitner et al., 2023). The ZooTraits could serve as an entry point for identifying these gaps, thereby stimulating new avenues for enhancing trait data collection. Nonetheless, the support of open‐source apps and centralized hubs, such as the Open Trait Network, has the potential to transform trait‐based ecology. We encourage users to contribute with trait information via the FeedTrait functionality, as it will enable us to build a global community to freely and effectively share trait information for multiple taxonomic groups within the Animal Kingdom.

In conclusion, we hope that the ZooTraits app will become a valuable and dynamic resource for ecology and evolution. The capability of exploring and comparing trait data across the animal kingdom provides researchers with a tool that will facilitate generalizations across groups and regions. Furthermore, by encouraging users to contribute trait information, ZooTraits seeks to bridge knowledge gaps and foster a global community of scientists to advance trait‐based ecology. As we move forward, the continued integration of this app with established frameworks (Damour et al., 2018; Gallagher et al., 2021; Gonçalves‐Souza et al., 2023; Keller et al., 2023; Palacio et al., 2022; Rosado et al., 2013, 2016; Weiss & Ray, 2019) will contribute to a more predictive trait‐based animal ecology.

## AUTHOR CONTRIBUTIONS

**Thiago Gonçalves‐Souza:** Conceptualization (lead); data curation (lead); formal analysis (lead); funding acquisition (equal); investigation (lead); methodology (lead); project administration (lead); software (lead); visualization (lead); writing – original draft (lead). **Beatriz Milz:** Data curation (lead); formal analysis (lead); resources (lead); software (lead); visualization (lead). **Nathan J. Sanders:** Conceptualization (supporting); writing – review and editing (equal). **Peter B. Reich:** Conceptualization (supporting); funding acquisition (lead); writing – review and editing (equal). **Brian Maitner:** Conceptualization (supporting); data curation (lead); writing – review and editing (equal). **Leonardo S. Chaves:** Data curation (equal); methodology (equal); writing – review and editing (equal). **Gabriel X. Boldorini:** Data curation (equal); methodology (equal); writing – review and editing (equal). **Natália Ferreira:** Data curation (equal); methodology (equal); writing – review and editing (equal). **Reginaldo A. F. Gusmão:** Data curation (equal); methodology (equal); writing – review and editing (equal). **Phamela Bernardes Perônico:** Data curation (equal); methodology (equal); writing – review and editing (equal). **Fabrício B. Teresa:** Data curation (equal); methodology (equal); writing – review and editing (equal). **María Natalia Umaña:** Conceptualization (lead); methodology (lead); writing – original draft (lead).

### OPEN RESEARCH BADGES

This article has earned an Open Data badge for making publicly available the digitally‐shareable data necessary to reproduce the reported results. The data is available at https://github.com/thiago‐goncalves‐souza/zootraits.

## Supporting information


Figure S1.

## Data Availability

All code and data are available on GitHub: https://github.com/thiago‐goncalves‐souza/zootraits.
