# Supplementary material for: ZooTraits: An R shiny app for exploring animal trait data for ecological and evolutionary research
Source: Ecol Evol. 2024 Apr 29;14(5):e11334. doi: 10.1002/ece3.11334 (PMC11056955; doi:10.1002/ece3.11334)
Supplement: Supplementary file 1 — Figure S1. [file ECE3-14-e11334-s001.docx]

**Support Information**

**ZOOTRAITS: AN R SHINY APP FOR EXPLORING ANIMAL TRAIT DATA FOR ECOLOGICAL AND EVOLUTIONARY RESEARCH**

THIAGO GONÇALVES-SOUZA^1,2,3*^, BEATRIZ MILZ^4^, NATHAN J. SANDERS^2^, PETER B. REICH^1,5^, BRIAN MAITNER^6^, LEONARDO S. CHAVES^7^, GABRIEL X. BOLDORINI^3^, NATÁLIA FERREIRA^8^, REGINALDO A. F. GUSMÃO^3^, PHAMELA BERNARDES PERÔNICO^9,10^, FABRÍCIO B. TERESA^9, 10^, MARÍA NATALIA UMAÑA^2^

1. Institute for Global Change Biology, School for Environment and Sustainability, University of Michigan, Ann Arbor, Michigan.
2. Department of Ecology and Evolutionary Biology, University of Michigan, Ann Arbor, Michigan.
3. Programa de Pós-Graduação em Etnobiologia e Conservação da Natureza, Departmento de Biologia, Universidade Federal Rural de Pernambuco, Recife, Brasil
4. Pós-graduação em Ciência Ambiental, Instituto de Energia e Ambiente, Universidade de São Paulo
5. Department of Forest Resources, University of Minnesota, St Paul, MN 55108
6. Department of Geography, University at Buffalo, Buffalo, New York
7. Comparative BioCognition, Institute of Cognitive Science, University of Osnabrück, Osnabrück, Germany
8. Programa de Pós-Graduação em Biodiversidade, Departmento de Biologia, Universidade Federal Rural de Pernambuco, Recife, Brasil.
9. Programa de Pós-Graduação em Recursos Naturais do Cerrado, Universidade Estadual de Goiás, Anápolis, Brasil.
10. Laboratório de Biogeografia e Ecologia Aquática, Universidade Estadual de Goiás, Anápolis, Brasil.

* Correspondence: [tgoncalv@umich.edu](mailto:tgoncalv@umich.edu)

**Table S1.** List of tools, resources, and websites to check taxonomic nomenclature. Users may also refer to Grenié et al. (2023) for a review on tools and databases for taxon names.

**R Packages**

- AmphiNom <<https://github.com/hcliedtke/AmphiNom>>
- bdc <<https://cloud.r-project.org/web/packages/bdc/index.html>>
- gatoRs <<https://cran.r-project.org/web/packages/gatoRs/index.html>>
- taxize < <https://cran.r-project.org/web/packages/taxize/taxize.pdf>>

**Encyclopedia**

- Catalogue of Life <<https://www.catalogueoflife.org/>>
- Encyclopedia of Life (EOL) <<https://eol.org/>>
- Integrated Taxonomic Information System <<https://www.itis.gov/>>
- Open tree of Life (TOL) <<https://tree.opentreeoflife.org/opentree>>
- Wikipedia list of taxonomy databases <<https://en.m.wikipedia.org/wiki/Category:Online_taxonomy_databases>>

**Taxon-specific websites**

- Amphibian database <<https://amphibiansoftheworld.amnh.org/>>
- Animal database <<https://zoobank.org/>>
- Ant database <<https://www.antweb.org/>>
- Bee database <<http://moure.cria.org.br/>>
- Bird database <<https://birdsoftheworld.org/bow/home>>
- Fish database <<https://www.fishbase.us/>>
- Mammal database <<https://www.mammaldiversity.org/>>
- Marine species database <<https://www.marinespecies.org/index.php>>
- Nematode database <<https://wormbase.org/>>
- Odonate database <<https://www.odonatacentral.org/app/#/wol/>>
- Paleobiology database <<https://paleobiodb.org/>>
- Primate database <<http://www.primate-sg.org/taxonomy/>>
- Reptile database <<http://reptile-database.org>>
- Scorpion database <<https://www.ntnu.no/ub/scorpion-files/intro.php>>
- Spider database <<https://wsc.nmbe.ch/>>
- Shark database <<https://shark-references.com/>


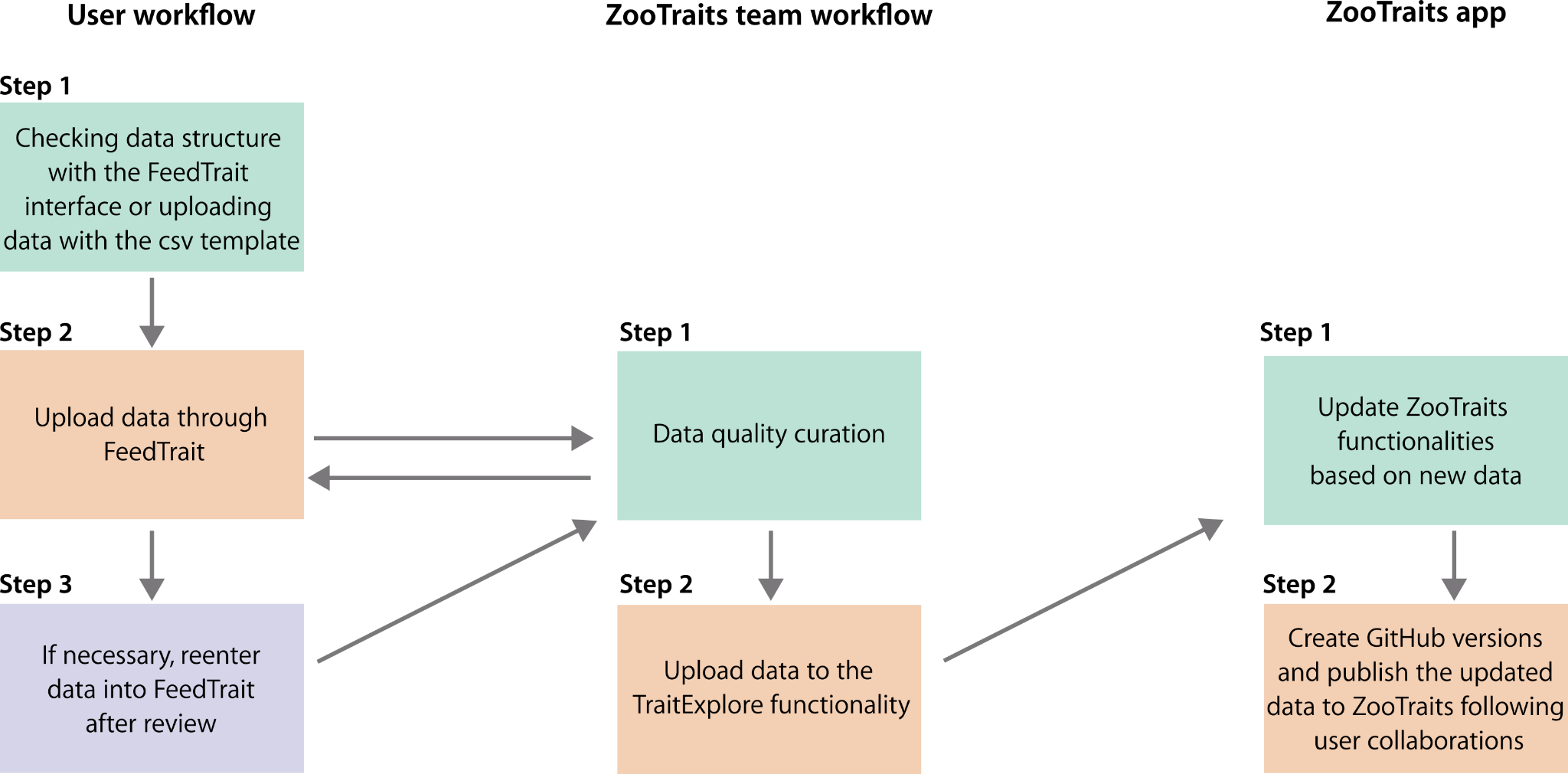


**Figure S1.** Quality assurance / quality control (QA/QC) workflow (user → ZooTraits team → ZooTraits app) for the FeedTrait functionality, which includes three steps from users (step 3 is necessary only if the step 1 - quality control - asks for data review from the user), quality assessment and upload from the ZooTraits team, and updates of the ZooTraits app and version control on GitHub.
